# Personality as a mediator of autistic traits and internalizing symptoms in two community samples

**DOI:** 10.1186/s40359-022-00774-z

**Published:** 2022-03-28

**Authors:** Olivia N. Grella, Amanda Dunlap, Alycia M. Nicholson, Kimberly Stevens, Brian Pittman, Silvia Corbera, Gretchen Diefenbach, Godfrey Pearlson, Michal Assaf

**Affiliations:** 1 Olin Neuropsychiatry Research Center, Institute of Living, Hartford Hospital, 200 Retreat Avenue, Hartford, CT 061016 USA; 2 Department of Psychiatry, Yale University School of Medicine, New Haven, CT USA; 3 Anxiety Disorders Center, Institute of Living, Hartford Hospital, Hartford, CT USA; 4 Department of Psychological Science, Central Connecticut State University, New Britain, CT USA

**Keywords:** Personality, Social impairment, Internalizing disorders, Gender, Autism spectrum disorder, Depression, Anxiety, Stress

## Abstract

**Background:**

Autism spectrum disorder (ASD) is characterized by deficits in social functioning and is comorbid with internalizing disorders and symptoms. While personality is associated with these symptoms and social functioning in non-ASD samples, its role mediating the relationship between ASD traits and internalizing symptoms is not clear.

**Methods:**

We studied the mediating effect of personality on the correlations between ASD traits and internalizing symptoms (i.e., depression, anxiety, stress) in two samples. Additionally, we explored the moderating effect of gender. Analyses were applied to a small (Study 1; *N* = 101) undergraduate sample. A broader sample recruited via an online crowdsourcing platform (Study 2; *N* = 371) was used to validate the results.

**Results:**

Study 1’s mediation analyses revealed that neuroticism was the only significant mediator. Study 2 replicated these results by finding extraversion to be an additional mediator for anxiety and extraversion, openness, and agreeableness as additional mediators for stress. Moderation analyses revealed that gender was never a significant moderator.

**Conclusions:**

These results support the effects of personality on the relationship between autism traits and internalizing symptoms. Future research should explore these effects in clinical samples to better understand the role of personality in symptomatology and the need to address it as part of intervention.

**Supplementary Information:**

The online version contains supplementary material available at 10.1186/s40359-022-00774-z.

## Background

Autism spectrum disorder (ASD), is a heterogeneous neurodevelopmental disorder with core deficits in social functioning [1]. Autism-related social deficits are often conceptualized as a spectrum, or a dimensional trait, lying across healthy and clinical populations with individuals diagnosed with ASD being on the extreme end [11]. Importantly, variation in ASD traits in non-clinical populations share similar genetic etiology with clinically diagnosed ASD [7, 30]. As individuals with ASD diagnosis show high rates of psychiatric comorbidities, including depression and anxiety [6, 27, 29], elevated autistic traits or social dysfunction, such as social withdrawal, are also associated with increased psychiatric symptomatology in individuals without an ASD diagnosis [30, 37].

Depression is one of the most common comorbid diagnoses of ASD [6, 29]. It has been suggested that there is a bi-directional relationship between the social impairments associated with ASD and depression [6]. Feeling uncomfortable in social situations might increase feelings of lack of inclusion or a lack of understanding how to behave, which can lead to avoidance behaviors that increase feelings of loneliness, a precursor to depression [6]. On the other hand, symptoms of depression may increase patients’ chances of poor social encounters. Another important factor is social awareness [41]. Those with high functioning ASD or those who are better at reading social situations are more likely to develop depression [41]. Additionally, subthreshold depression (i.e., elevated levels of depressive symptoms without meeting the full diagnostic criteria for major depression disorder) is known to have similar risk factors as a clinically diagnosed depressive disorder [15]. Thus, non-clinical individuals with high depressive symptoms may experience similar impairments in social situations and awareness.

Similarly, anxiety disorders are also common in ASD. Generalized anxiety disorder (GAD), social phobias, and social anxiety disorder (SAD) are strongly associated with social impairment [37]. In a study that investigated the role of anxiety in the development of ASD symptoms, 50% of the participants with a dual diagnosis of ASD and SAD expressed the significance that social impairment had on the prevalence and maintenance of SAD symptoms [33]. Another study [41] found a similar relationship when studying GAD. Importantly, subthreshold anxiety has been known to predict worse social functioning in comparison to healthy controls [26]. As in depression, anxiety could have a bi-directional relationship with social impairment.

Thus, there is a great degree of heterogeneity in psychiatric comorbidity in ASD that can be partially explained by the severity of the individuals’ social impairments and their insight. Another potentially important factor is personality. Personality reflects differences in cognitive processing, emotion recognition, and behavioral styles on an individual level and is known to strongly predict psychiatric symptoms and diagnoses [43]. Although personality traits can be described and quantified in different ways, the Five-Factor Model (FFM) of personality developed by McCrae and Costa (1987) has extensive research supporting its use in a variety of cultures, languages, and samples [34, 43]. The FFM includes five dimensional traits: neuroticism, extraversion, openness to experience (referred to as openness in the remainder of this paper), agreeableness, and conscientiousness.

As mentioned, extensive research has demonstrated that personality traits, conceptualized as continuums as well, are related to internalizing symptoms in both clinical and general populations [24]. Importantly, research has supported the notion that personality traits are not only predictors, but also provide a theoretical basis for psychopathology [16, 24]. It has been theorized that different levels of the five personality traits impacts the presence and severity of varying mental health symptoms. For example, neuroticism has been consistently shown to predict anxiety and depression in both clinical and non-clinical samples [25, 40]. In relation to anxiety, those higher in neuroticism tended to be more aware of anxiety and stress responses which heightened their reactions to those situations [22, 42].

Regarding the other personality traits, extraversion correlated negatively with self-reported internalizing symptoms [23]. Individuals who were more extraverted were less likely to develop anxiety or depression. Openness, while not studied among mental health often, tends to be explored by its subdomains. For example, openness to actions negatively correlated while openness to fantasy positively correlated with depression in a nonclinical sample [9]. Openness to action also served as a significant predictor of psychological well-being as it was negatively correlated with both depression and anxiety [9]. Studies of the agreeableness FFM trait have indicated that low agreeableness can amplify stress and lead to negative mental health symptoms in clinical samples [36, 43]. While studies have shown that agreeableness is related to externalizing disorders more than internalizing disorders [36], it has also been shown to have a significant impact on depression and anxiety [25]. Lastly, conscientiousness is believed to indirectly influence depression, anxiety, and stress [36]. This trait has been shown to increase the vulnerability to internalizing disorders as low scores can increase problems in daily functioning [40]. On the other hand, higher scores on conscientiousness may decrease this risk because of the ability to self-regulate emotions.

The relationship between personality and ASD diagnoses and related-symptoms is not clear due to the relative scarcity of studies. Although a recent meta-analysis [31] found that neuroticism was positively associated with ASD diagnoses and traits while extraversion, openness, agreeableness, and conscientiousness had a negative association, the authors emphasized variability in personality profiles. Importantly, variation in personality traits was related to functional outcome in this population [39, 44].

While personality, internalizing symptoms, and ASD traits, specifically social functioning, are interconnected, research on all three variables is limited. In studying the role of social support and social conflict as a mediator between personality and psychological distress, Finch and Graziano (2001) found that the association between personality traits and psychological distress, specifically between neuroticism, extraversion, agreeableness, and depression, was mediated by negative perception of social support and social conflict. Smith et al. (2017) focused on the role of neuroticism and conscientiousness as moderators on the relationship between social withdrawal and internalizing disorders in adolescents. Surprisingly, neuroticism was not a moderator for depression or anxiety and conscientiousness was only a moderator for depression. The authors concluded that increased conscientiousness in teens with higher levels of social withdrawal might be a protective factor from increased depressive symptoms due to improved self-regulation.

Given the limited literature, additional research is need to determine how personality traits mediate the relationship between ASD traits and related social impairments and internalizing symptoms. Identifying mediators impacting the effect of social dysfunction on psychiatric symptoms may inform treatment targets for improving emotional resilience in patients with ASD. As an initial step toward this goal, the current study aimed to depict these interactions in community samples which represent a range of ASD, internalizing, and personality traits. The temporal order of the variables was based off of the previous literature, such as Smith et al. (2017), which suggested that personality traits may have a moderating effect between social withdrawal and internalizing symptoms. As social withdrawal is a type of social dysfunction, a primary deficit of ASD, it was decided to follow this temporal order when studying the mediating effects of these variables. It was hypothesized that personality would have a mediating effect on the correlations between ASD traits and internalizing symptoms. However, the exact nature of these mediating effects was not predicted due to the limited information available.

Our secondary goal was to test the moderating effect of gender on positive mediation effects. Gender differences are demonstrated in ASD, personality, and internalizing symptoms. Although ASD is more common in males, it has been shown that the distribution of autistic traits across the general population is even across genders [11]. On the other hand, traits are often less severe and less prevalent in women [11]. In regard to psychopathology, women are twice as likely as men to be diagnosed with depression and are more likely to be diagnosed with GAD, specific phobias, and panic disorder with and without agoraphobia [13, 38]. As for personality, there is a minimal (10%) overlap in men’s and women’s personality profiles [38]. Men score significantly lower than women on neuroticism, agreeableness, and conscientiousness [38]. They also scored lower on extraversion and openness, but not significantly. Taken together, it is important to consider the role gender has on the mediating effect of personality on the association between ASD traits and internalizing symptoms. It was hypothesized that there would be a greater gender moderation effect in females based on the current literature suggesting that women tend to score higher on personality traits and internalizing symptoms when compared to men.

Two samples were recruited to achieve the study’s goals. The first consisted of a relatively small (*N* = 101) undergraduate student sample, used to delineate the mediating and moderating effects described above in an exploratory fashion, due to the limited knowledge available. The second sample included participants from a crowdsourcing platform (MTurk; see below) that was used to validate the findings of Study 1 in a larger (*N* = 371), broader, community sample.

## Study 1: undergraduate student sample

### Methods

#### Participants

Participants for Study 1, referred to as the Student Sample, were recruited from four undergraduate institutions in Connecticut and Massachusetts (Central Connecticut State University, Trinity College, University of Hartford, and Bay Path University) through undergraduate psychology, research, and neuroscience courses. Two hundred and thirty-six undergraduate students consented into the study, but only 127 completed all assessments and passed the quality assurance (QA) criteria of the study (see below). Nineteen students were additionally excluded due to intelligence quotient (IQ) scores on the Abbreviated 9-Item Raven’s Progressive Matrices Test (“Complete the Pattern”; RSPM) of the fifth percentile or less. Six participants were excluded due to contradicting information provided regarding their psychiatric history. One final participant was also excluded due to an extreme outlier score on the Depression Anxiety and Stress Scale-21 (DASS-21) depression subscale. Thus, 101 undergraduate students (36 males and 65 females) were included in the final analyses of this study. The average age of the sample was 19.95 (*SD* = 3.25). The majority of the sample identified as Caucasian (82.20%) and non-Hispanic/Latino (83.20%). For further details refer to Table 1.

**Table 1:** Sample characteristics and groups comparisons

	Student *N* = 101	MTurk *N* = 371	Group Statistics	*p*
Age (years)	19.95 ± 3.25	38.49 ± 11.67	*t*(470) = 15.79	< 0.0001*
Gender (M/F)	36/65	160/210^	χ^2^(2) = 2.16	0.34
Race (C/AA/A/O/NR)	83/10/3/4/1	315/30/22/1/3	χ^2^(5) = 13.72	0.02*
Ethnicity (H/NH/NR)	12/84/5	26/341/4	χ^2^(2) = 9.26	0.01*
Abbreviated Raven’s IQ (percentile)	30.43 ± 21.32	38.18 ± 23.98	*t*(470) = 2.95	0.003*
Neuroticism (T score)	55.76 ± 11.44	49.33 ± 15.08	*t*(470) = − 3.99	< 0.0001*
Extraversion (T score)	53.38 ± 11.07	46.92 ± 14.43	*t*(470) = − 4.17	< 0.0001*
Openness (T score)	52.56 ± 9.94	56.15 ± 12.02	*t*(470) = 2.75	0.01*
Agreeableness (T score)	51.44 ± 10.61	51.59 ± 14.44	*t*(470) = 0.10	0.92
Conscientiousness (T score)	48.05 ± 10.54	53.10 ± 11.89	*t*(470) = 3.88	< 0.0001*
DASS-Depression	9.05 ± 10.45	7.42 ± 10.10	*t*(470) = − 1.42	0.16
DASS-Anxiety	9.88 ± 8.73	4.70 ± 6.64	*t*(470) = − 6.48	< 0.0001*
DASS-Stress	12.38 ± 9.41	8.75 ± 8.58	*t*(470) = − 3.68	< 0.0001*
SRS Total (T score)	55.17 ± 8.15	53.99 ± 10.50	*t*(470) = − 1.05	0.30

M, Male; F, Female; C, Caucasian; AA, African American; A, Asian; O, Other; NR, Not Reported; H, Hispanic/Latino(a); NH, Non-Hispanic/Latino(a)

^*^ *p* < 0.05

^ one participant did not report gender

### Materials

The *Social Responsiveness Scale-2* (SRS-2) is a self-report measure used to quantify autistic traits, with an emphasis on social behaviors [12]. The 65-item questionnaire was answered on a scale from 1 (*not true*) to 4 (*almost always true*). Participants received a total score which was then computed into a t-score. Higher scores indicate a higher level of symptom severity and higher social impairment. The SRS-2 has consistently displayed excellent reliability with alphas ranging from 0.94 to 0.96 [8]. It has also been reported to have good content, predictive, and construct validity. The current study also had good internal consistency with an alpha of 0.93.

The *Depression Anxiety Stress Scales-21* (DASS-21) was used to assess depression, anxiety, and stress states [32]. It contained 21 questions rated on a Likert scale from 0 (*did not apply to me at all*) to 3 (*applied to me very much, or most of the time*). Participants received subscores for depression, anxiety, and stress. Higher scores on each scale indicated more severe symptoms. The current study had good internal consistency for depression (α = 0.92), anxiety (α = 0.80), and stress (α = 0.88). Between scales correlations were higher in this sample than previously reported. The current sample had *r*’s of 0.84 for depression and anxiety, 0.82 for depression and stress, and 0.81 for anxiety and stress. Previously reported values have been 0.46, 0.57, and 0.72, respectively [2].

The *NEO Five-Factor Inventory-3* (NEO-FFI-3*)* measures participants’ placement on the five traits of personality: neuroticism/emotional stability, extroversion, openness to experience, agreeableness, and conscientiousness [35]. 60 self-report questions were answered on a Likert scale ranging from 0 (*strongly agree*) to 4 (*strongly disagree*). A score was received on each of the five personality traits. Higher scores indicated how strongly participants aligned with the personality trait. The NEO-FFI-3 has displayed good internal consistency with ranges from 0.68 to 0.86 [35]. The current sample had a similar range of 0.65 (openness)—0.88 (neuroticism). Extraversion (α = 0.80), conscientiousness (α = 0.83), and agreeableness (α = 0.71) fell towards the middle of the range. It is important to note though, that the FFM was reported to be both appropriate and reliable in ASD samples [21].

The revised *Abbreviated 9-Item Raven’s Progressive Matrices Test* (“Complete the Pattern”; RSPM) was used as a non-verbal measure of intelligence (see Additional file 1: Supplement #1 for further information) [5]. Participants are required to complete patterns with multiple-choice answer options. Scores on the abbreviated version are a reliable predictor of scores on the full version [5]. Participants received a raw score which also gets converted into a percentile score based on their age.

### Procedure

Participants in the student sample were invited to sign up for the study through courses and research announcements at their institution. Those who signed up were directed to a REDCap [18, 19] survey where all the measures were located. Participants indicated their agreement to participate through an electronic consent form at the start of the survey and confirmed they were older than 18 years’ old, US residents, and fluent in English. Participants then completed questions regarding their demographics as well as medical and psychiatric history. Next, they completed a series of questionnaires and tasks, among them the SRS-2, DASS-21, NEO-FFI-3, and the RSPM, reported here. The RSPM was only used as an inclusion criterion. Participants scoring at or below the fifth percentile were excluded from analyses. The assessments included quality control questions to check for consistent answers (5% repeated questions) and to make sure the participants were attentive to the tasks (5% “catch items” designed as simple questions that all participants should be able to answer correctly). Participants with less than 75% of the QA questions answered correctly and that completed the study protocol in less than 60% of the anticipated time were excluded from data analyses. Upon protocol completion, students were given course credit for their research participation. All study procedures were approved by Hartford Healthcare Institutional Review Board (IRB) and the IRBs of the recruiting institutions.

### Statistical analysis

**Fig. 1:**
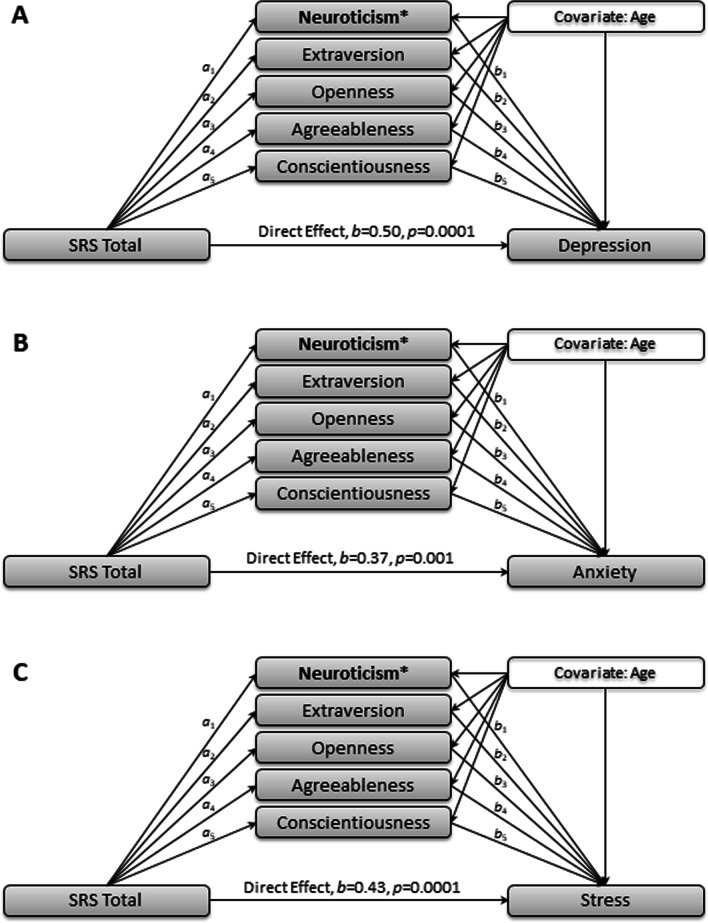
Mediations models from Study 1 with significant indirect effects indicated with an asterisk (*). See Table 2  for complete effect values for for *a* and *b* paths as well as the indirect effects (a*b)

Three multiple mediator models were used to examine the direct and indirect effects of personality traits on the association between autism traits, as measured by the SRS, and internalizing symptoms (DASS-21). Each multiple mediator model was conducted using model 4 (test of multiple mediation) of the PROCESS macro v3 for SPSS [20]. SRS total scores were included as the independent variable, personality traits as the five potential mediating variables, and internalizing symptoms (i.e., one of three DASS-21 subscales) as the dependent variable. Age was included as a covariate in the three models (see Fig. 1 for a visual and Table 2 for *a* and *b* paths, indirect (a*b) effects, and their confidence intervals; the direct effect, *c’*, is stated in the text below). The PROCESS macro estimates both direct and indirect effects using percentile-based 5000 bootstrapped 95% confidence intervals (CI). A CI that does not include zero reflects a significant direct or indirect effect. Pairwise comparisons were next used to denote the relative impact of each mediating variable on the overall model. A confidence interval that does not include zero suggests a significant difference in the relative indirect effect of one proposed mediating variable over another.

**Table 2:** Study 1 mediation analyses

	*a* path					*b* path					Indirect effect a*b			
	Effect	*SE*	*t*	95% CI		Effect	*SE*	*t*	95% CI		Effect	*SE*	95% CI	
				LL	UL				LL	UL			LL	UL
*Outcome = Depression*														
1. Neuroticism	0.79*	0.12	6.72	0.55	1.02	0.42*	0.08	5.36	0.26	0.57	0.33**	0.08	0.19	0.48
2. Extraversion	− 0.55*	− 0.13	− 4.38	− 0.80	− 0.30	− 0.09	0.07	− 1.24	− 0.24	0.06	0.05	0.05	− 0.03	0.15
3. Openness	0.08	0.12	0.68	− 0.16	0.33	− 0.02	0.08	− 0.26	− 0.17	0.13	− 0.002	0.01	− 0.03	0.02
4. Agreeableness	− 0.58*	0.12	− 4.94	− 0.82	− 0.35	0.12	0.08	1.57	− 0.03	0.28	− 0.07	0.05	− 0.16	0.06
5. Conscientiousness	− 0.53*	0.12	− 4.47	− 0.77	− 0.30	− 0.04	0.08	− 0.55	− 0.21	0.12	0.02	0.05	− 0.07	0.12
*Outcome = Anxiety*														
1. Neuroticism	0.79*	0.12	6.72	0.55	1.02	0.38*	0.07	5.60	0.24	0.51	0.30**	0.06	0.18	0.43
2. Extraversion	− 0.55*	− 0.13	− 4.38	− 0.80	− 0.30	0.05	0.06	0.82	− 0.07	0.18	− 0.03	0.03	− 0.10	0.04
3. Openness	0.08	0.12	0.68	− 0.16	0.33	− 0.02	0.07	− 0.35	− 0.16	0.11	− 0.002	0.01	− 0.03	0.03
4. Agreeableness	− 0.58*	0.12	− 4.94	− 0.82	− 0.35	0.09	0.07	1.31	− 0.05	0.22	− 0.05	0.05	− 0.16	0.05
5. Conscientiousness	− 0.53*	0.12	− 4.47	− 0.77	− 0.30	− 0.08	0.07	− 1.14	− 0.22	0.06	0.04	0.05	− 0.05	0.14
*Outcome = Stress*														
1. Neuroticism	0.79*	0.12	6.72	0.55	1.02	0.40*	0.07	5.90	0.26	0.53	0.31**	0.07	0.19	0.47
2. Extraversion	− 0.55*	− 0.13	− 4.38	− 0.80	− 0.30	0.04	0.06	0.65	− 0.09	0.18	− 0.02	0.03	− 0.09	0.04
3. Openness	0.08	0.12	0.68	− 0.16	0.33	0.11	0.07	1.60	− 0.03	0.24	0.01	0.02	− 0.03	0.05
4. Agreeableness	− 0.58*	0.12	− 4.94	− 0.82	− 0.35	− 0.01	0.07	− 0.20	− 0.15	0.12	0.01	0.05	− 0.07	0.15
5. Conscientiousness	− 0.53*	0.12	− 4.47	− 0.77	− 0.30	− 0.01	0.07	− 0.17	− 0.15	0.13	0.01	0.04	− 0.08	0.10

The *a* path refers to the effect of SRS scores and the NEO-FFI-3. The *b* path refers to the effect of the NEO-FFI-3 and the DASS-21 subscales

^*^ *p* ≤ 0.05; **Significant mediators, based on confidence intervals [CI] without 0

Three additional models were run to test if gender moderated the above mediation models (one for each DASS-21 subscale as the dependent variable). PROCESS v3 model 8 was used in these analyses, which allows for moderated mediation. Gender was included as a moderator for autistic traits and personality (*a* path) and autistic traits and depression (*c* path). Age was included as a covariate, SRS total as the independent variable, and a DASS-21 subscale as the dependent variable in respective analyses.

### Results

All analyses were conducted using SPSS version 26.0 and PROCESS macro for SPSS version 3.5 [20]. Before running the mediation and moderation analyses, correlations between the SRS total *t*-score and the five personality traits and three subscales of the DASS-21 were run. This was to assure that there was a general association between the variables before continuing with the main analyses. Pearson *r* correlations revealed significant correlations between the SRS and neuroticism (*r*(99) = 0.57, *p* < 0.001), extraversion (*r*(99) = -0.41, *p* < 0.001), agreeableness (*r*(99) = − 0.45, *p* < 0.001), and conscientiousness (*r*(99) = -0.42, *p* < 0.001). The correlation between SRS and openness was not significant (*p* = 0.55). As for the DASS-21, Pearson *r* correlations revealed significant results between the SRS and depression (*r*(99) = 0.63, *p* < 0.001), anxiety (*r*(99) = 0.59, *p* < 0.001), and stress (*r*(99) = 0.64, *p* < 0.001). Fisher *r*-to-*z* comparison between group correlations indicated no significant differences between genders, with the exception of agreeableness (see Additional file 2: Supplemental #2 for further details).

Next, age was correlated with all study variables to see if it should be controlled for in further analyses. Pearson *r* correlations revealed no significant relationships between age and all study variables (*p* > 0.01). However, after running correlations between age and the study variables in Study 2 and finding several significant relationships, it was decided to add age as a covariate in this sample as well.

### Mediation of personality traits

*Depression.* The overall model predicting depression was significant, *F*(7, 93) = 18.47, *p* < 0.001, and explained 58.16% of the variance in depression scores. The direct effect (*c’* path) of autistic traits on depression was significant, *b* = 0.50, *SE* = 0.12, *t* = 4.07, *p* < 0.001. The total effect model (i.e., the direct and indirect effect of autism traits on depression controlling for age) was also significant, *F*(2, 98) = 34.93, *p* < 0.001. Of the *a* paths, autism traits significantly predicted neuroticism, extraversion, agreeableness, and conscientiousness, but not openness. Results from the *b* paths indicated that only neuroticism predicted depression.

Bootstrapped confidence intervals indicated that the indirect effect of neuroticism was significant. There were no significant indirect effects of extraversion, openness, agreeableness, or conscientiousness. Pairwise comparisons suggest that the indirect effect of neuroticism was significantly greater than that of extraversion, openness, agreeableness, and conscientiousness. There were no significant differences in the indirect effects among extraversion, openness, agreeableness, and conscientiousness.

*Anxiety.* The overall model predicting anxiety was significant, *F*(7, 93) = 15.86, *p* < 0.001, and explained 54.41% of the variance in anxiety. The direct effect of autism traits on anxiety was significant, *b* = 0.37, *SE* = 0.11, *t* = 3.48, *p* < 0.001. The total effect model was also significant, *F*(2, 98) = 26.03, *p* < 0.001. Of the *a* paths, autism traits significantly predicted neuroticism, extraversion, agreeableness, and conscientiousness, but not openness. Results from the *b* paths indicated that only neuroticism predicted anxiety.

Bootstrapped confidence intervals indicated that the indirect effect of neuroticism was significant. There were no significant indirect effects of extraversion, openness, agreeableness, or conscientiousness. Pairwise comparisons suggest that the indirect effect of neuroticism was significantly greater than that of extraversion, openness, agreeableness, and conscientiousness. There were no significant differences in the indirect effects among extraversion, openness, agreeableness, and conscientiousness.

*Stress.* The overall model predicting stress was significant, *F*(7, 93) = 20.82, *p* < 0.001, and explained 61.05% of the variance in stress. The direct effect of autism traits on stress was significant, *b* = 0.43, *SE* = 0.11, *t* = 4.08, *p* < 0.001. The total effect model was also significant, *F*(2, 98) = 34.58, *p* < 0.001. Of the *a* paths, autism traits significantly predicted neuroticism, extraversion, agreeableness, and conscientiousness, but not openness. Results from the *b* paths indicated that only neuroticism predicted stress.

Bootstrapped confidence intervals indicated that the indirect effect of neuroticism was significant. There were no significant indirect effects of extraversion, openness, agreeableness, or conscientiousness. Pairwise comparisons suggest that the indirect effect of neuroticism was significantly greater than that of extraversion, openness, agreeableness, and conscientiousness. There were no significant differences in the indirect effects among extraversion, openness, agreeableness, and conscientiousness.

#### Gender moderation

*Depression.* While the outcome model was significant, *F*(9, 91) = 14.31, *p* < 0.0001, and explained 58.60% of the variance in depression, gender was not a significant moderator across FFM subscales, *F*(1,91) = 0.14, *p* = 0.71.

*Anxiety.* While the outcome model was significant, *F*(9, 91) = 12.41, *p* < 0.0001, and explained 55.10% of the variance in anxiety, gender was not a significant moderator across FFM subscales, *F*(1,91) = 1.02, *p* = 0.32.

*Stress.* Again, while the outcome model was significant, *F*(9, 91) = 16.09, *p* < 0.0001, and explained 61.41% of the variance in stress, gender was not a significant moderator across FFM subscales, *F*(1,91) = 0.06, *p* = 0.80.

### Discussion

The purpose of Study 1 was to test the potential mediating effects of personality traits on the significant associations between autistic traits and depression, anxiety, and stress in a small sample of undergraduate college students. Secondly, the possibility of gender as a moderator of the significant mediations was examined. Overall, only neuroticism was a mediator in all three models. Specifically, neuroticism was a significant mediator when individuals reported higher ASD traits and higher scores of depression, anxiety, or stress. Finally, gender was not a significant moderator in any of these mediations.

Since this was an exploratory study due to the limited nature of the research available, it is difficult to draw direct parallels from the current literature. However, because neuroticism is the personality trait that has been most consistently shown to be related to emotional stability [34], it is logical to conclude that this trait would impact the relationship between ASD traits and internalizing symptoms. Our results are in agreement with previous studies demonstrating that the more emotionally stable a person is, the less likely they are to experience various mental health symptoms and to have difficulties in social situations [22, 40, 42]. What was surprising is that gender was not a significant moderator in any of these mediations. This may be due to a few factors. For one, this was a relatively small sample which might not have had the power to show significant small or medium effects. Also, while autistic traits are more commonly diagnosed in males rather than females [11], depression and anxiety are more commonly diagnosed in females [13, 38]. These contrasting gender effects might have masked its potential effects on these results.

Given these limitations, and the narrow characteristics of the college student sample, to validate the results presented in Study 1, we conducted Study 2 that included a larger more diverse sample.

## Study 2: crowdsourcing Amazon MTurk sample

### Methods

This study utilized Amazon Mechanical Turk (MTurk), which is a crowdsourcing platform, to recruit a wider sample of participants across the United States. Sample size estimates were conducted using G*Power Version 3.1.9.2 a priori power analysis [14] with 80% power and 5% error probability. A previous study found that there was a small to medium effect of openness on modern health worries and neuroticism (Cohen’s *f* = 0.10) [42]. Thus, Cohen’s *f* = 0.10 was used to calculate the sample size necessary for finding a similar effect in the current study. The results indicated that a sample size of 144 was necessary in order to detect a small-to-medium effect (Cohen’s *f* = 0.10). Although this method has been commonly used in psychology and psychiatry research in recent years due to its effectiveness in gathering large scale datasets quickly [17], it is important to note that MTurk samples have reported different findings than traditional recruitment [10]. Specifically, they reported to be more open and lower on extraversion, agreeableness, and neuroticism.

### Participants

Study 2 utilized a community crowd sourced sample. Seven hundred and eighty four participants consented into the study through MTurk (MTurk sample). Only 613 successfully completed the protocol. One hundred and thirty eight participants were excluded due to not passing the QA questions and completing the protocol in less than 36 min. Lastly, three participants were excluded due to conflicting medical or psychiatric information, 10 were excluded for having severe neurological conditions, and 85 participants were excluded due to scores on the RSPM. Additionally, six participants were excluded due to extreme scores on the DASS-21 anxiety subscale. Thus, 371 adults (160 males, 210 females, and 1 not reported) were included in the final MTurk sample. Participants had a mean age of 38.49 (*SD* = 11.67). A majority of the sample identified as Caucasian (84.90%) and non-Hispanic/Latino (91.90%). Additional details, as well as group differences between both samples, are detailed in Tables 1 and 3.

**Table 3:** Group characteristics by gender

	Student				MTurk			
	Males *n* = 36	Females *n* = 65	Group Statistics	*p*	Males *n* = 160	Females *n* = 210	Group Statistics	*p*
Age	20.69 ± 4.04	19.54 ± 2.67	*t*(99) = − 1.73	0.09	37.66 ± 11.40	39.19 ± 11.84	*t*(368) = 1.25	0.21
Neuroticism	49.53 ± 11.58	59.22 ± 9.85	*t*(99) = 4.44	< 0.0001*	45.69 ± 14.31	51.98 ± 15.06	*t*(368) = 4.07	< 0.0001*
Extraversion	51.97 ± 9.68	54.15 ± 11.76	*t*(99) = 0.95	0.35	49.59 ± 14.38	44.82 ± 14.15	*t*(368) = − 3.19	0.002*
Openness	51.06 ± 9.21	53.40 ± 10.30	*t*(99) = 1.14	0.26	55.05 ± 11.51	56.90 ± 12.32	*t*(368) = 1.47	0.14
Agreeableness	49.67 ± 11.52	52.42 ± 10.03	*t*(99) = 1.25	0.21	48.86 ± 14.41	53.69 ± 14.18	*t*(368) = 3.22	0.001*
Conscientiousness	47.25 ± 9.46	48.49 ± 11.14	*t*(99) = 0.57	0.57	53.97 ± 11.25	52.54 ± 12.28	*t*(368) = − 1.15	0.25
DASS-Depression	7.56 ± 10.17	9.88 ± 10.59	*t*(99) = 1.07	0.29	6.95 ± 10.47	7.73 ± 9.82	*t*(368) = 0.74	0.46
DASS-Anxiety	7.83 ± 8.58	11.02 ± 8.67	*t*(99) = 1.77	0.08	3.83 ± 6.09	5.34 ± 6.98	*t*(368) = 2.19	0.03*
DASS-Stress	10.47 ± 10.66	13.43 ± 8.55	*t*(99) = 1.52	0.13	7.46 ± 8.08	9.72 ± 8.85	*t*(368) = 2.56	0.01*
SRS	55.14 ± 8.24	55.18 ± 8.15	*t*(99) = 0.03	0.98	53.56 ± 10.28	54.31 ± 10.69	*t*(368) = 0.68	0.50

^*^p < 0.05

### Materials

Participants in Study 2 completed the same measures as those in Study 1. These measures include the SRS-2, DASS-21, NEO-FFI-3, and the RSPM. The SRS-2 still displayed excellent internal consistency with an alpha of 0.96. The DASS-21 also had good internal consistency for depression (α = 0.94), anxiety (α = 0.86), and stress (α = 0.89). Intercorrelations were lower than the student sample. Depression and anxiety (α = 0.66), depression and stress (α = 0.67), and anxiety and stress (α = 0.72) were all closer to previously reported values from Antony et al. (1998). The NEO-FFI-3 had a higher range than Study 1 of internal consistency from 0.78 (openness) to 0.92 (neuroticism), with extraversion (α = 0.87), agreeableness (α = 0.84), and conscientious (α = 0.87) showing higher internal consistencies as well.

### Procedure

As in Study 1, participants in the MTurk sample were invited to sign up for the study conducted on REDCap [18, 19] through the MTurk platform. They followed the same study procedure and needed to meet the same inclusion criteria as in Study 1. Quality control measures were also included throughout the assessments as described above. Upon protocol completion, MTurk participants received $5 compensation for their research participation (only if passing QA). All study procedures were approved by Hartford Healthcare IRB.

### Statistical analysis

**Fig. 2:**
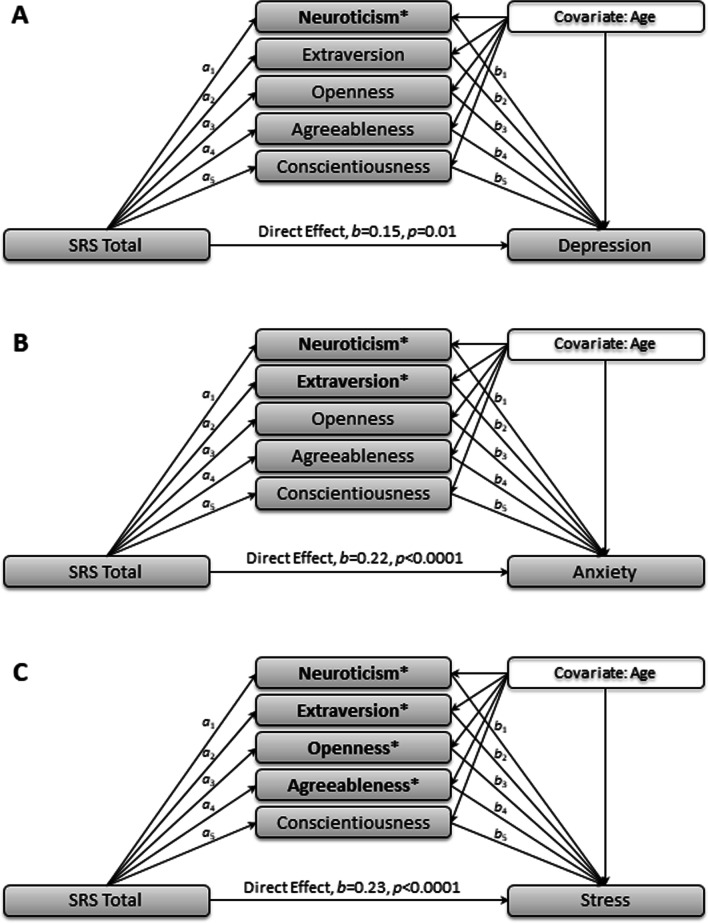
Mediations models from Study 2 with significant indirect effects indicated with an asterisk (*). See Table 3 for complete effect values for *a* and *b* paths as well as the indirect effects (a*b)

Three multiple mediator models were again used to examine the direct and indirect effects of personality traits on the association between autism traits, as measured by the SRS, and internalizing symptoms (DASS-21). SRS total scores were included as the independent variable, personality traits as the five potential mediating variables, and internalizing symptoms (i.e., one of three DASS-21 subscales) as the dependent variable. Age was included as a covariate in the three models (see Fig. 2 and Table 4 for direct effects, indirect effects, and CIs).

**Table 4:** Study 2 mediation analysis

	*a* path					*b* path					Indirect effect a*b			
	Effect	*SE*	*t*	95% CI		Effect	*SE*	*t*	95% CI		Effect	*SE*	95% CI	
				LL	UL				LL	UL			LL	UL
*Outcome = Depression*														
1. Neuroticism	0.97*	0.06	17.16	0.86	1.08	0.36*	0.04	9.86	0.29	0.43	0.35**	0.04	0.27	0.43
2. Extraversion	− 0.84*	0.06	− 13.90	− 0.96	− 0.72	− 0.07*	0.03	− 2.27	− 0.13	− 0.01	0.06	0.03	− 0.0004	0.12
3. Openness	− 0.13*	0.06	− 2.03	− 0.25	− 0.004	0.05	0.03	1.63	− 0.01	0.11	− 0.01	0.01	− 0.20	0.001
4. Agreeableness	− 0.74*	0.06	− 12.02	− 0.87	− 0.06	0.05	0.03	1.73	− 0.01	0.11	− 0.04	0.03	− 0.09	0.01
5. Conscientiousness	− 0.56*	0.05	− 10.73	− 0.67	− 0.46	− 0.05	0.04	− 1.18	− 0.12	0.03	0.03	0.02	− 0.02	0.07
*Outcome = Anxiety*														
1. Neuroticism	0.97*	0.06	17.16	0.86	1.08	0.20*	0.03	7.57	0.15	0.25	0.20**	0.03	0.14	0.25
2. Extraversion	− 0.84*	0.06	− 13.90	− 0.96	− 0.72	0.10*	0.02	4.14	0.05	0.14	− 0.08**	0.03	− 0.13	− 0.03
3. Openness	− 0.13*	0.06	− 2.03	− 0.25	− 0.004	0.03	0.02	1.15	− 0.02	0.07	− 0.003	0.004	− 0.01	0.002
4. Agreeableness	− 0.74*	0.06	− 12.02	− 0.87	− 0.06	− 0.01	0.02	− 0.50	− 0.06	0.03	0.01	0.02	− 0.03	0.05
5. Conscientiousness	− 0.56*	0.05	− 10.73	− 0.67	− 0.46	0.03	0.03	1.12	− 0.02	0.09	− 0.02	0.02	− 0.06	0.02
*Outcome = Stress*														
1. Neuroticism	0.97*	0.06	17.16	0.86	1.08	0.29*	0.03	9.78	0.23	0.35	0.28**	0.03	0.22	0.35
2. Extraversion	− 0.84*	0.06	− 13.90	− 0.96	− 0.72	0.07*	0.03	2.83	0.02	0.12	− 0.06**	0.03	− 0.11	− 0.01
3. Openness	− 0.13*	0.06	− 2.03	− 0.25	− 0.004	0.09*	0.03	3.64	0.04	0.14	− 0.01**	0.01	− 0.03	− 0.001
4. Agreeableness	− 0.74*	0.06	− 12.02	− 0.87	− 0.06	− 0.11*	0.03	− 4.24	− 0.16	− 0.06	0.08**	0.02	0.04	0.13
5. Conscientiousness	− 0.56*	0.05	− 10.73	− 0.67	− 0.46	0.03	0.03	0.91	− 0.03	0.09	− 0.01	0.02	− 0.06	0.02

The *a* path refers to the effect of SRS scores and the NEO-FFI-3. The *b* path refers to the effect of the NEO-FFI-3 and the DASS-21 subscales

^*^ *p* ≤ 0.05; **Significant mediators, based on confidence intervals [CI] without 0

Additional models were run to test if gender moderated the above mediation models, as described above for Study 1. Gender was included as a moderator for autistic traits and personality (*a* path) and autistic traits and depression (*c* path). Age was included as a covariate. This same model was used in the additional gender moderations with the DASS-21 subscale changing.

### Results

The analyses for Study 2 followed the methods and data analytic plan from Study 1. Correlation analyses between the SRS and the five personality traits and the three subscales from the DASS-21 revealed significant relationships with neuroticism (*r*(369) = 0.69, *p* < 0.001), extraversion (*r*(369) = -0.58, *p* < 0.001), agreeableness (*r*(369) = -0.56, *p* < 0.001), and conscientiousness (*r*(369) = -0.52, *p* < 0.001) as well as depression (*r*(369) = 0.59, *p* < 0.001), anxiety (*r*(369) = 0.54, *p* < 0.001), and stress (*r*(369) = 0.63, *p* < 0.001). The correlation between the SRS and openness was nonsignificant (*p* = 0.12). Fisher *r*-to-*z* comparison between group correlations indicated no significant differences between genders (see Additional file 2: Supplemental #3 for further details).

Next, age was correlated with all study variables. Age was found to be significantly correlated with neuroticism (*r*(369) = -0.26, *p* < 0.001), agreeableness (*r*(369) = 0.22, *p* < 0.001), conscientiousness (*r*(369) = 0.21, *p* < 0.001), depression (*r*(369) = -0.22, *p* < 0.001), anxiety (*r*(369) = -0.25, *p* < 0.001), stress (*r*(369) = -0.24, *p* < 0.001), and the SRS (*r*(369) = -0.29, *p* < 0.001). Due to multiple significant associations, age was entered as a covariate in all analyses.

#### Mediation of personality traits

*Depression.* The overall model predicting depression was significant, *F*(7, 363) = 58.66, *p* < 0.001, and explained 53.08% of the variance in depression. The direct effect of autism traits on depression was significant, *b* = 0.15, *SE* = 0.06, *t* = 2.66, *p* < 0.001. The total effect model was also significant, *F*(2, 368) = 93.19, *p* < 0.001. Of the *a* paths, autism traits significantly predicted all five personality traits. Results from the *b* paths indicated that neuroticism and extraversion predicted depression, but openness, conscientiousness, and agreeableness were not significant.

Bootstrapped confidence intervals indicated that only the indirect effects of neuroticism were significant. Pairwise comparisons suggest that the indirect effect of neuroticism was significantly greater than that of extraversion, openness, agreeableness, and conscientiousness. In addition, the indirect effect of extraversion was significantly less than that of openness and agreeableness. There were no significant differences in the indirect effects among extraversion, openness, agreeableness, and conscientiousness.

*Anxiety.* The overall model predicting anxiety was significant, *F*(7, 363) = 36.61, *p* < 0.001, and explained 41.39% of the variance in anxiety. The direct effect of autism traits on anxiety was significant, *b* = 0.22, *SE* = 0.04, *t* = 5.18, *p* < 0.001. The total effect model was also significant, *F*(2, 368) = 78.17, *p* < 0.001. Of the *a* paths, autism traits significantly predicted all five personality traits. Results from the *b* paths indicated that neuroticism and extraversion predicted anxiety, but openness, conscientiousness, and agreeableness were not significant.

Bias corrected bootstrapped confidence intervals indicated that the indirect effects of neuroticism and extraversion were significant. However, there were no significant indirect effects of openness, conscientiousness, and agreeableness. Pairwise comparisons suggest that the indirect effect of neuroticism was significantly greater than that of extraversion, openness, agreeableness, and conscientiousness. In contrast, the indirect effect of extraversion was significantly lower than that of openness, agreeableness, and conscientiousness. There were no significant differences in the indirect effects among openness, agreeableness, and conscientiousness.

*Stress.* The overall model predicting stress was significant, *F*(7, 363) = 67.75, *p* < 0.001, and explained 56.65% of the variance in stress. The direct effect of autism traits on stress was significant, *b* = 0.23, *SE* = 0.05, *t* = 4.82, *p* < 0.001. The total effect model was also significant, *F*(2, 368) = 122.57, *p* < 0.001. Of the *a* paths, autism traits significantly predicted all five personality traits. Results from the *b* paths indicated that neuroticism, extraversion, openness, and agreeableness predicted stress, but conscientiousness was not significant.

Bootstrapped confidence intervals indicated that the indirect effects of neuroticism, extraversion, openness, and agreeableness were significant. There was no significant indirect effect of conscientiousness. Pairwise comparisons suggest that the indirect effect of neuroticism was significantly greater than that of extraversion, openness, agreeableness, and conscientiousness. In contrast, the indirect effect of extraversion was significantly less than that of openness and agreeableness. There were no significant differences in the indirect effects among openness, agreeableness, and conscientiousness.

#### Gender moderation

*Depression.* While the outcome model was significant, *F*(9, 360) = 47.82, *p* < 0.0001, and explained 54.45% of the variance in depression, gender was not a significant moderator across FFM subscales, *F*(1, 360) = 0.04, *p* = 0.83.

*Anxiety.* While the outcome model was significant, *F*(9, 360) = 29.02, *p* < 0.0001, and explained 42.05% of the variance in anxiety, gender was not a significant moderator across FFM subscales, *F*(1, 360) = 2.38, *p* = 0.12.

*Stress.* Again, while the outcome model was significant, *F*(9, 360) = 53.52, *p* < 0.0001, and explained 57.23% of the variance in stress, gender was not a significant moderator across FFM subscales, *F*(1, 360) = 1.41, *p* = 0.24.

### Discussion

The aim of Study 2 was to validate the results of Study 1 in a larger, broader, community sample. Overall, the results were replicated along with additional significant mediators. While neuroticism was a significant mediator in all three models as in Study 1, additional personality traits were significant mediators in two out of the three models. Anxiety scores were mediated by both neuroticism and extraversion, such that higher scores of neuroticism and lower scores of extraversion significantly mediated the positive correlation between ASD traits and anxiety. For the stress model, neuroticism, extraversion, openness, and agreeableness were all significant mediators. Specifically, higher scores of neuroticism and lower scores of extraversion, openness, and agreeableness significantly mediated the relationship between ASD traits and stress.

As mentioned, the role of neuroticism as a mediator between ASD traits and internalizing symptoms is the most consistent result both between our two studies and the different symptom models (i.e., depression, anxiety, and stress). As mentioned earlier, this result highlights the importance of emotional stability, as reflected by the neuroticism trait, in mediating the relationships between ASD traits and internalizing symptoms [22, 40, 42]. It is less clear why people who are less extraverted (i.e., more introverted) are more likely to experience higher levels of social difficulties alongside anxiety and stress, but not depression. Also, it is less clear why individuals who are less open and less agreeable are more likely to experience higher level of stress alongside ASD traits/social difficulties, but not depression or anxiety. Extraversion is associated with how sociable and outgoing individuals are [34], while introversion is often associated with anxiety and fewer social interactions [23]. It can be speculated that individuals who are more introverted and anxious alongside having higher impairment in social functioning would be associated with more distress. This is similar for feelings of stress as well, as one source of stress can be social situations or interacting with others. However, we speculate that individuals who score higher on symptoms of depression might not be impacted by extraversion because they do not have the desire to interact with others. It is not that they are too nervous to do so; it is just that they are not willing to engage in these situations to begin with.

In the third model where stress was the outcome, agreeableness and openness were additional mediators. Agreeableness is generally associated with altruistic behaviors and openness is described as originality and curiosity to novel experiences [34]. Taken at face value, these traits seem to have little relationship to internalizing symptoms, but they do have social natures to them. Generally speaking, the results indicate that being less agreeable can lead to a stronger relationship between poor social functioning and heightened stress. We suggest that the less willing someone is to help others can lead to more difficulties in social situations and possibly increase their level of stress. Similarly, being less open to new experiences can also make someone less likely to engage in social situations and can increase their stress when placed into them.

Lastly, like in Study 1, gender was not a significant moderator for any of the mediation models. Thus, even with a larger sample size, it seems that gender does not moderate the mediation effects of personality on the relationship between ASD traits and depression, anxiety, and stress. As mentioned above, this could be due to the previous research that suggests an opposite effect of gender on ASD traits, with men being more likely to score higher on the SRS [11], and with women being more likely to score higher on depression, stress, and anxiety traits [13, 38].

## Conclusions

Study 1 and Study 2 both highlighted the mediating effects that neuroticism has on the relationship between ASD traits and the internalizing symptoms of depression, anxiety, and stress. Study 2 extended the results of Study 1 by finding additional mediations, possibly due to the larger sample size and a more diverse sample. While neuroticism was the only mediator for the depression model, extraversion was an added mediator for the anxiety model and extraversion, openness, and agreeableness were added mediators for the stress model. Overall, gender was never a moderator of any mediation effects in either Study 1 or Study 2.

The results of both studies add to the current research that studies the relationship between ASD traits, mainly social functioning, and internalizing disorder symptoms [16, 40]. Most relevant to our results and as mentioned in the introduction, Smith et al. (2017) were investigating the moderating effects of neuroticism and conscientiousness on the relationship between social withdrawal and internalizing disorders and indicated a potential role that personality plays. While neuroticism was not a moderator for depression or anxiety, conscientiousness was a moderator for depression. The current study expanded on these results by suggesting a mediation effect instead and focusing on three self-reported states of depression, anxiety, and stress. While conscientiousness was not a mediator in the current study, neuroticism was consistently found to be one. We speculate that these additional mediators might be related to the group differences in Study 1 and Study 2 (see Table 3). In accordance with previous studies that showed increased rate of depression, anxiety and stress in undergraduate samples [3, 4], we found higher levels on the anxiety and stress in Study 1’s sample, and these were the two outcomes that demonstrated additional mediators. It could be that higher levels of anxiety and stress are not impacted by personality, with the exception of neuroticism. Therefore, more research is needed to better understand this relationship, including in clinical samples.

These results should be interpreted with some limitations in mind. The data were collected online without real-time in-person monitoring. Data collected specifically through MTurk have been shown to obtain slightly different results on personality measures compared to traditional methods of study completion [10]. Although we implemented rigorous QA procedures, our results might have been biased by these methods. Future studies should attempt to use in-person data collection measures to replicate and validate our results. Another limitation is that we used mediation analyses to test the cross-sectional data in this study. While mediation analyses tend to be associated with longitudinal research to assess causal hypotheses, the current study was exploratory and did not aim to find causal relationships. Additionally, using mediation analyses with cross-sectional data has been supported [20]. Future research would benefit from replicating our results in a longitudinal study. Also, a self-reported ASD trait questionnaire, the SRS, was used to measure autistic traits and approximate related social abilities. While this measure was originally developed as a screening tool for ASD and potentially as a measure of ASD symptom severity, it has been validated in community samples [11]. While the SRS measures both social functioning and, to a lesser degree, additional autistic traits such as repetitive behaviors and restricted interests, its manual specifically notes that higher scores are an indicator of difficulties in social functioning. Future studies should expand the measures used to assess different aspects of social functioning. Another limitation is that both samples were a majority female (64% in Study 1 and 57% in Study 2) and they had higher scores on some DASS-21 and NEO subscales in both studies. This could have driven the lack of moderation effects on the mediation effects between autistic traits and internalizing symptoms. Future research should better balance male and female recruitment. A final limitation is that self-report measures were used to collect information on internalizing symptoms and are not indicative of clinical diagnoses. Comparing our results to clinical samples is warranted.

In light of these limitations, this study may suggest that personality plays a bigger role in the relationship between social dysfunction associated with ASD traits and internalizing symptoms than known before. This is in accord with the emphasis given in recent years to the role of personality in the development and maintenance of psychiatric symptoms and thus to its importance in designing treatment protocols [28, 45]. Specifically, understanding the role of neuroticism might help better explain the presentation of ASD traits and internalizing symptoms in different clinical and non-clinical populations. Since autistic traits in non-clinical samples and ASD diagnosis share common etiological and genetic similarities [7, 30], our results can have implications for clinical samples. More specifically, if these results are validated in clinically diagnosed ASD samples, they can have implications for current ASD interventions and the need to target certain personality traits, such as neuroticism.

## Supplementary Information

**Additional file 1**. Revised model of the abbreviated nine-item form of the *Raven's Standard Progressive Matrices*.

**Additional file 2**. Fisher *r*-to-*z* transformations for Study 1 and Study 2.

## Data Availability

The data that support the findings of this study are available from the corresponding author upon reasonable request.

## Abbreviations

ASD: Autism spectrum disorder
DASS: Depression Anxiety and Stress Scale
FFM: Five-factor model
GAD: Generalized anxiety disorder
IQ: Intelligence quotient
IRB: Institutional Review Board
MTurk: Amazon Mechanical Turk
NEO-FFI: NEO Five-Factor Inventory
QA: Quality assurance
RSPM: Raven’s progressive matrices test
SAD: Social anxiety disorder
SRS: Social Responsiveness Scale
