# Supplementary material for: Personality as a mediator of autistic traits and internalizing symptoms in two community samples
Source: BMC Psychol. 2022 Mar 28;10:81. doi: 10.1186/s40359-022-00774-z (PMC8962582; doi:10.1186/s40359-022-00774-z)
Supplement: Supplementary file 1 — Additional file 1. Revised model of the abbreviated nine-item form of the Raven's Standard Progressive Matrices. [file 40359_2022_774_MOESM1_ESM.docx]

Personality as a Mediator of Autistic Traits and Internalizing Symptoms in Two Community Samples

Olivia N. Grella, M.A.

Amanda Dunlap, M.S.

Alycia M. Nicholson, B.S.

Kimberly Stevens, Ph.D.

Brian Pittman, M.S.

Silvia Corbera, Ph.D.

Gretchen Diefenbach, Ph.D.

Godfrey Pearlson, M.D.

Michal Assaf, M.D.

**Supplementary #1: Assessment of General Intelligence with the Abbreviated Nine-Item Form of the Raven’s Standard Progressive Matrices**

The Raven’s Standard Progressive Matrices (RSPM) assesses fluid intelligence and has been shown to be an indicator of general intelligence (Raven, 2000). The original test is comprised of 5 sets of 12 black and white pattern matching tasks, with increasing difficulty across sets. The main limitation of this task is its length and abbreviated versions have been developed. Scores on the abbreviated version are a reliable predictor of full version scores (Bilker et al., 2012). In the current study we administered a variant of the abbreviated nine-item form developed by Bilker and colleagues (2012). The nine-item version strongly predicts the full 60-item RSPM score, while cutting the administration time by more than 75%. In the current study, two items were administered incorrectly owing to obtaining the items from an earlier version of the measure. Specifically, items I11 and I55 in the original form were replaced by items I12 and I58 in the current study, respectively. Importantly, the replacement items were from the same difficulty set as the original items (sets A and E, respectively). Upon uncovering the error we sought consultation and Dr. Bilker provided us with a modified predictive model, to be used with the items included in our study, as outlined below:

Revised Model for the current study:

Rˆ=exp(1.103 + 0.106*I12 + 0.180*I24 + 0.235*I28 + 0.096*I36 + 0.454*I43 + 0.455*I48 + 0.252*I49 + 0.476*I53+ 0.305*I58),

V=I12 + I24 + I28 + I36 + I43 + I48 + I49 + I53 + I58, and the predicted scale total of Ŷ = 60 – (V +R ^) (Ŷ is increased to 0 if negative).

For reference, the original Model for Form A is provided below:

Rˆ=exp(1.323 + 0.198*I11 + 0.216*I24 + 0.237*I28 + 0.142*I36 + 0.374*I43 + 0.304*I48 + 0.178*I49 + 0.458*I53+ 0.289*I55),

V=I11 + I24 + I28 + I36 + I43 + I48 + I49 + I53 + I55, and the predicted scale total of Ŷ = 60 – (V +R ^)

To explore the validity of this adapted version Dr. Bilker computed correlations of the revised model derived scores with the full RSPM scores as described for the original nine-item abbreviated version (Bilker et al., 2012). Results demonstrated high correlations for both the modeling and validation datasets (r=0.97078 and 0.92303, respectively), which are similar to those reported for the original derived scores based on the model for form A (r=0.98361 and 0.90634). These correlations support comparability of the standard nine-item abbreviated version and the version used in the current study.

Finally, for our data Ŷ  scores were transformed to percentile norm scores based on age, using the RSPM Percentile Norms for Adults in the USA table (Raven, Raven, & Court, 2000). Participants with a percentile score of 5 or lower were excluded from analyses.

References:

Bilker, W. B., Hansen, J. A., Brensinger, C. M., Richard, J., Gur, R. E., & Gur, R. C. (2012). Development of abbreviated nine-item forms of the Raven's standard progressive matrices test. *Assessment, 19*(3), 354-369. doi:10.1177/1073191112446655

Raven, J. (2000). The Raven’s Progressive Matrices: Change and stability over culture and time. *Cognitive Psychology, 41*, 1-48.

Raven, J., Raven, J. C., & Court, J. H. (2000). *Standard Progressive Matrices Raven Manual: Section 3* (2000 ed.). San Antonia, TX: Pearson.
