# Supplementary material for: Personality as a mediator of autistic traits and internalizing symptoms in two community samples
Source: BMC Psychol. 2022 Mar 28;10:81. doi: 10.1186/s40359-022-00774-z (PMC8962582; doi:10.1186/s40359-022-00774-z)
Supplement: Supplementary file 2 — Additional file 2. Fisher r-to-z transformations for Study 1 and Study 2. [file 40359_2022_774_MOESM2_ESM.docx]

Personality as a Mediator of Autistic Traits and Internalizing Symptoms in Two Community Samples

Olivia N. Grella, M.A.

Amanda Dunlap, M.S.

Alycia M. Nicholson, B.S.

Kimberly Stevens, Ph.D.

Brian Pittman, M.S.

Silvia Corbera, Ph.D.

Gretchen Diefenbach, Ph.D.

Godfrey Pearlson, M.D.

Michal Assaf, M.D.

Supplementary #2: Correlations with the SRS Total T-Scores by Gender in the Student Sample

| Correlations with SRS | Males & Females Combined | Males | Females | *p* † |
| --- | --- | --- | --- | --- |
| NEO Neuroticism (T score) | 0.57** | 0.70** | 0.57** | 0.31 |
| NEO Extraversion (T score) | -0.41** | -0.23 | -0.50** | 0.14 |
| NEO Openness (T score) | -0.08 | -0.01 | -0.09 | 0.71 |
| NEO Agreeableness (T score) | -0.45** | -0.74** | -0.27* | 0.002 |
| NEO Conscientiousness (T score) | -0.42** | -0.41* | -0.42** | 0.96 |
| DASS-Depression | 0.63** | 0.66** | 0.62** | 0.75 |
| DASS-Anxiety | 0.59** | 0.56** | 0.62** | 0.67 |
| DASS-Stress | 0.64** | 0.67** | 0.64** | 0.81 |

**Correlation is significant at the 0.01 level. *Correlation is significant at the 0.05 level. †Difference

between males and females correlation by using Fisher r-to-z transformation.

Supplementary #3: Correlations with the SRS Total T-Scores by Gender in the MTurk Sample

| Correlations with SRS | Males & Females Combined | Males | Females | *p* † |
| --- | --- | --- | --- | --- |
| NEO Neuroticism (T score) | 0.69** | 0.74** | 0.68** | 0.25 |
| NEO Extraversion (T score) | -0.58** | -0.58** | -0.58** | 1.00 |
| NEO Openness (T score) | -0.06 | -0.10 | -0.07 | 0.78 |
| NEO Agreeableness (T score) | -0.56** | -0.59** | -0.56** | 0.67 |
| NEO Conscientiousness (T score) | -0.52** | -0.59** | -0.48** | 0.14 |
| DASS-Depression | 0.59** | 0.55** | 0.60** | 0.48 |
| DASS-Anxiety | 0.54** | 0.50** | 0.57** | 0.35 |
| DASS-Stress | 0.63** | 0.62** | 0.64** | 0.75 |

**Correlation is significant at the 0.01 level. †Difference between males and females correlation by using

Fisher r-to-z transformation.
